# Open-Label-Placebos can reduce pain, but not indigestion during gluten challenge in chronic pain patients

**DOI:** 10.3389/fpsyg.2025.1572761

**Published:** 2025-06-04

**Authors:** Lena Paschke-Dahl, Regine Klinger

**Affiliations:** Department of Anesthesiology, University Medical Center Hamburg-Eppendorf, Hamburg, Germany

**Keywords:** gluten intolerance, chronic pain, expectation, Open-Label-Placebo, fibromyalgia

## Abstract

**Background:**

Dietary interventions have become a management tool for chronic pain conditions over the past few decades. Certain diets, such as gluten-free diets, are perceived as particularly beneficial by patients, although there is no evidence to support this. Studies that have investigated this topic have focused little on possible expectation effects that could be involved in symptom development or pain increase.

**Methods:**

In a 2×2 study design with repeated measurements to test treatment effects, we investigated 26 patients with fibromyalgia (FMS). Additional chronic pain conditions were included and analyzed exploratively. However, the main analysis focused on fibromyalgia patients. Participants underwent an oral food challenge (OFC) with double-blinded gluten or alleged gluten (sham gluten). All of them received an OLP with different instructions to treat negative effects of the porridge. Treatment expectations were modulated by either neutral or positive instructions regarding the OLPs. Participants were randomly assigned to one of four groups: (1) gluten and neutral instructions; (2) gluten and positive instructions; (3) sham gluten and neutral instructions; and (4) sham gluten and positive instructions. Expectations before (T0) and after the instructions (T0.1) as well as pain and indigestion before (T0) and after the OFC (T1 30min, T2 30-180min, T3 240min) were assessed.

**Results:**

In FMS patients, a significant interaction with instructions were observed (*p* = 0.048). Positive instructions led to a decrease in pain (T0-T2) while neutral instructions led to an increase in pain. However, *post-hoc* comparisons did not reveal significant group differences. No interaction was found with gluten (*p* = 0.65). Positive instructions increased positive treatment expectations but missed significance marginally (*p* = 0.06), while negative expectations decreased for all participants regardless of instructions (*p* < 0.001). A strong correlation was found between expected and actual pain relief (*p* < 0.001). Digestive discomfort increased temporarily post-intervention (*p* < 0.004) but returned to baseline after 4 h. No significant effects of gluten (*p* = 0.15) or instructions (*p* = 0.8) on indigestion were observed.

**Conclusion:**

This study highlights the complex interplay of disease type, placebo effects, and expectations in chronic pain conditions during gluten provocation. While gluten itself showed no significant impact on pain or indigestion, positive instructions significantly enhanced perceived pain relief. These findings suggest that expectation effects, rather than gluten, may play a more central role in symptom modulation, at least for pain. Future research should focus on expectation-driven mechanisms to better understand and optimize dietary interventions in chronic pain management and differences across pain diseases.

## 1 Introduction

Chronic pain, with a prevalence of 12–30%, affects many individuals and significantly impairs their quality of life (Breivik et al., 2006). In recent years, dietary interventions have gained increasing attention in the management of chronic pain conditions (Brain et al., 2019; Mazza et al., 2023; Field et al., 2021; Kaushik et al., 2020; Lu et al., 2023; Elma et al., 2022), and a healthy nutrition is assumed to be beneficial for managing chronic pain conditions (Bautista et al., 2024). Although no specific diet has been proven to be particularly beneficial for managing chronic pain (Field et al., 2021), certain dietary trends have emerged over the past few decades (Fang et al., 2023), particularly the gluten-free diet (Choung et al., 2016). Sales of gluten-free products have risen sharply in recent years, reflecting this trend (Statista, 2013). The development of symptoms following gluten exposure in the absence of abnormal biomarkers is now referred to as Non-Celiac Gluten Sensitivity (NCGS). However, the prevalence and reliability of this disorder is controversial (Reese et al., 2018), and following a gluten-free diet in the absence of a gluten-related disorder may be detrimental to health due to possible nutritional deficiencies of macronutrients and micronutrients (Diez-Sampedro et al., 2019).

Several chronic pain conditions have been examined in relation to gluten consumption, including fibromyalgia (FMS) (Garcia-Leiva et al., 2015; Isasi et al., 2015; Rodrigo et al., 2014), arthritis (Lidon et al., 2022), irritable bowel syndrome (IBS) (Rej and Sanders, 2018), endometriosis (Marziali et al., 2012) or headache (Martin and Vij, 2016). The present study originally aimed to investigate the role of expectation on the effect of gluten and Open-Label-Placebos (OLP) in a homogenous group of FMS patients. However, due to recruiting difficulties, the inclusion criteria were expanded to also allow patients with IBS and chronic headaches to participate. These diagnoses differ in their pathophysiology. Nevertheless, to date, no consistent evidence has emerged that a gluten-free diet leads to general improvement in any of these conditions (Lidon et al., 2022; Martin and Vij, 2016; Brouns et al., 2023; Almirall et al., 2022; Weaver and Herfarth, 2021), suggesting that multiple mechanisms, including psychological and contextual factors, may contribute. Furthermore, evidence suggests that there may be some overlap between pathophysiological mechanisms between some of the pain conditions. IBS and FMS, for instance, are associated with central sensitization (Brouns et al., 2023; Almirall et al., 2022; Weaver and Herfarth, 2021). Studies show a link between FMS and IBS, with 46–49% of people with FMS also having IBS (Harris and Johns, 2011; Locher et al., 2017). Similarly, women with endometriosis are more likely to get IBS (Daniali and Flaten, 2019), and several studies report co-occurrence between FMS and endometriosis (Daniali et al., 2024; Peerdeman et al., 2024; Younger et al., 2012; Schmitz et al., 2019), although findings are mixed (Klinger et al., 2017).

Despite these unclear similarities and differences in the pathophysiology, our sample shares an important psychological commonality. The key inclusion criterion across all sub-groups was the belief that gluten is a trigger for pain exacerbation, and this belief provided the basis for investigating expectancy-related mechanisms.

In this context the concept of placebo/nocebo effects is significant. It plays a critical role in dietary interventions (Harris and Johns, 2011). In their review, Neumann et al. (2022) showed that psychological factors are involved in dietary changes. Expectations are important in diet changes, as they not only include symptom improvement, but also health-promoting, weight-reducing effects and/or appreciation from the social environment. Public access to opinions of researchers, experts and food advertisements can shape these individual beliefs and expectations. Operant and classical conditioning processes are also part of the rationale for dietary change (Locher et al., 2017). An example of these mechanisms is the media-driven narrative around gluten, which can reinforce nocebo responses (Daniali and Flaten, 2019; Daniali et al., 2024). This is supported by a recent study by de Graaf et al. (2024) which found expectations of consuming gluten led to gastrointestinal symptoms in individuals with NCGS, even when no gluten was actually consumed.

A question arises from these findings: how can expectation effects be harnessed therapeutically? A promising approach is Open-Label-Placebo (OLP) treatment, in which patients receive an inert substance. These treatments have shown beneficial effects in IBS and chronic lower back pain (Younger et al., 2012; Schmitz et al., 2019; Klinger et al., 2017; Solle et al., 2021; Smits et al., 2021; Schaefer et al., 2018). Most studies that use OLPs provide detailed placebos to improve outcomes, though few investigate the effect of this information. For instance, Schaefer et al. (2018) did not find a higher placebo effect in allergic rhinitis participants who received information (Carvalho et al., 2016). However, they did not verify whether the extended information actually led to a change in expectations. In contrast, Locher et al. (2017) could find significant differences between the effectiveness of OLP with and without rationale (Locher et al., 2017). A qualitative review from Daniali and Flaten (2019) also found other non-specific factors such as confidence, professionalism, positive non-verbal behaviors (Daniali and Flaten, 2019) or the researcher’s expectation (Daniali et al., 2024) to result in lower pain reports and higher placebo effects, suggesting that also other factors influence these mechanisms.

The present study was conducted to test whether different forms of verbal information regarding OLP would lead to different treatment expectations and thus different treatment outcomes (pain and indigestion) in patients with fibromyalgia during a gluten provocation challenge. Our hypotheses were as follows: 1. Positive instructions lead to an increase of treatment expectation regarding OLP. 2. A high treatment expectation leads to better treatment outcomes regarding pain. 3. A high treatment expectation leads to better treatment outcomes regarding indigestion.

Furthermore, we conducted exploratory data analysis to gain further information on these hypotheses in the additional patient samples with chronic pain: irritable bowel syndrome and chronic headache.

## 2 Materials and methods

### 2.1 Participants

Patients 18 years or older with a diagnosis of fibromyalgia (FMS), irritable bowel syndrome (IBS), or headache (migraine or tension headache) were eligible to participate in the study. The study planned to include only fibromyalgia patients, but due to recruitment difficulties, other pain disorders linked to gluten were also included. The participants consisted of both patients who clearly believed they suffered from gluten intolerance and those who could imagine a possible association. Patients with cognitive impairments, insufficient knowledge of German, or a severe mental or physical disorder were excluded from the study. A further exclusion criterion comprised abnormal blood values indicating the presence of coeliac disease or gluten allergy. Relevant blood values were total IgA (0.4–3.5 g/L), specific IgE (wheat flour; <0.35 kU/l), and transglutaminase antibodies IgA (<7u/ml). To ensure a reliable blood analysis, participants had to confirm that they consumed gluten-containing foods during the last 3 months.

### 2.2 Study design

The aim of our randomized controlled clinical study was to investigate the role of instructions regarding OLP on patients’ expectations and thus on treatment results. A 2×2 full factorial, fully balanced within-subject and between-subject study design was used to examine the influence of instructions and gluten on symptom and pain development. Instruction (positive with the aim of high expectation, neutral with the aim of low expectation) and food challenge (sham gluten/real gluten) were fully crossed. The oral food challenge (OFC) included four measurement time points: before the OFC; 30 min after the OFC; 60–180 min after the OFC; and 4 h after OFC.

### 2.3 Expectation manipulation

**Figure 1:**
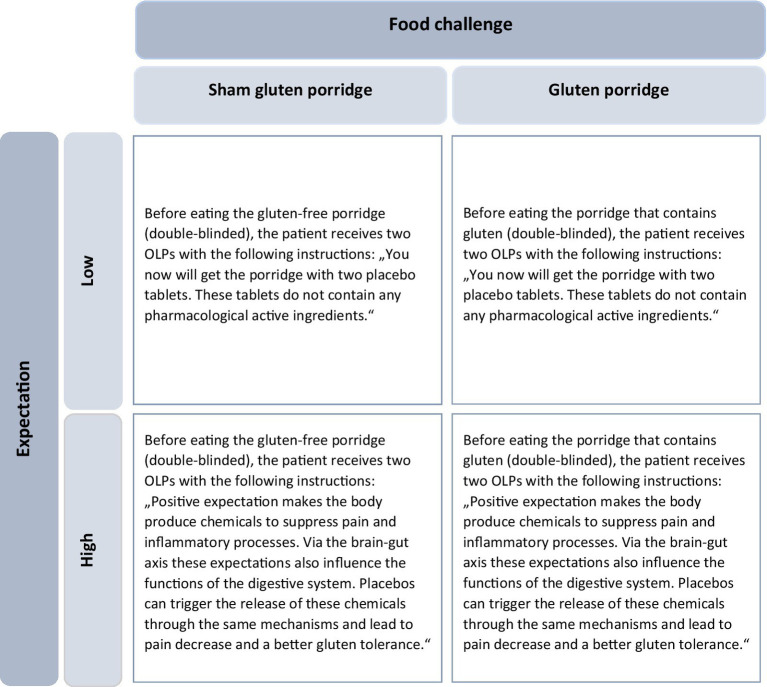
2×2 experimental design. Participants were divided into four groups according to expectations (low or high) and type of food (sham-gluten porridge or gluten porridge). The instructions for the OLP varied depending on the level of expectation (Adapted from Paschke et al., 2023, licensed under CC BY-NC 4.0). OLP, Open-Label-Placebo.

Expectation was modified by two different sets of instructions. Participants in the neutral group were only told that they would receive a pharmacologically active substance-free placebo, and they were given no explanation about mechanisms behind the OLP. In the positive group, the researcher gave a standardized rationale about possible psychophysiological processes behind the placebo effects of the OLP. These referred to both, pain mechanisms and gluten intolerance. With regard to the mechanisms underlying pain, the endogenous opioid system was elucidated using a comprehensible language. The role of expectation was emphasized. To positively modulate the expectation of gluten intolerance, the efficacy of OLPs was suggested by briefly describing their effects on the brain-gut axis. Again, expectation and other psychological factors were explained to be linked to the functions of the digestive system in this positive instruction. With these instructions, participants should form an expectation regarding pain and indigestions after eating the porridge (Figure 1).

The researcher was a female member of the research team who introduced herself as a psychologist. She wore a laboratory coat throughout the experiment. She administered the information about OLP, therefore no blinding was made regarding this group assignment.

### 2.4 Procedure/course of the study

#### 2.4.1 Phase 1: baseline

Interested patients were contacted via telephone and assessed for participation eligibility based on the inclusion criteria. If participation was considered, the patients received the first set of questionnaires and the informed-consent form. Baseline characteristics were assessed with German pain questionnaire including indication of the average pain on a numerical rating scale (NRS). Further, a questionnaire to determine general attitude toward medication (GAMQ (Peerdeman et al., 2024)) and gluten was included. Baseline expectation regarding OLP were assessed using the Stanford Expectations of Treatment Scale (SETS (Younger et al., 2012), zit, cf. “outcomes”). During the first screening appointment, the study physician conducted a detailed anamnesis and informed the patients about the general conditions of the study. After that, participants had to follow a gluten-free diet for 10 days for the following two reasons: (1) to emphasize the effect of the food challenge; and (2) to reinforce expectation effects for the upcoming food challenge, based on potential symptom improvement during the dietary change.

#### 2.4.2 Phase 2: oral food challenge

**Figure 2:**
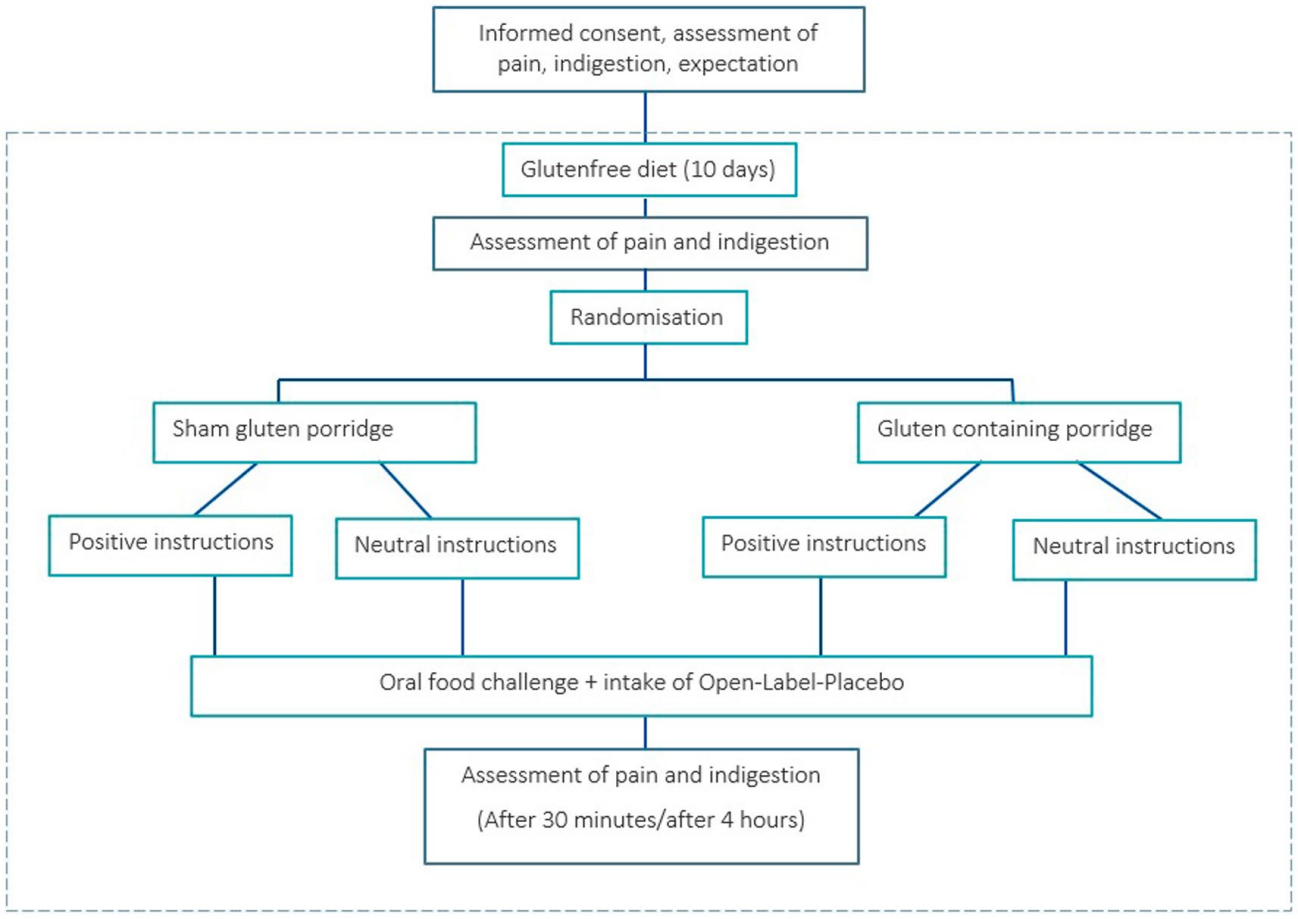
Flowchart of chronic pain patients undergoing an oral food challenge and Open-Label-Placebo intervention. (Adapted from Paschke et al., 2023, licensed under CC BY-NC 4.0).

Participants underwent an oral food challenge during a second in-house appointment. All participants were given an informed consent at the start of the study, explaining that they would be randomly assigned to receive either real or sham gluten (porridge with and without gluten) during the provocation. The first step of the oral food challenge was to document the current health status of each participant with regard to pain and indigestions. The patients were then randomly assigned to one of the four groups through the drawing of an envelope that was opened by the researcher. Randomization was stratified by age, sex, and disease. The envelope contained the information “neutral” or “positive” and the number 1 for “no gluten” or 2 for “gluten.” To ensure double-blinding regarding the porridge, the allocation of the numbers was unknown to the investigator, who gave the envelope to a second person who knew the meaning of the numbers. While the second person prepared the porridge, the researcher handed the placebos to the participants and gave either positive or neutral instructions (Figure 1). After that, participants had to rate again their expectation regarding the effects of the OLP (SETS). Each participant then took two placebo tablets, ate the porridge, and was taken to a holding room. The second measurement time point was 30 min after eating the porridge. The participants received the same questions regarding pain and potential indigestions. Subsequently, each participant received a list of these symptoms to document potential changes occurring between this first post-OFC measurement time point and the second one after 4 h. The participants were instructed not to eat anything in the meantime and to drink only water or unsweetened coffee or tea. If they desired, they could leave the building and pursue other activities, provided there were no contraindications (e.g., emerging nausea symptoms). They were asked to come back after 4 h for the last measurement time point. Again, they reported their current health status with regard to pain and indigestion (Figure 2 for whole flowchart).

### 2.5 Outcomes

Outcomes were tested with patient-related measures. The course of pain during the oral food challenge depicts the primary outcome. The measurement points were (1) before the oral food challenge; (2) 30 min after the oral food challenge; (3) 30–180 min after the oral food challenge; and (4) 4 h after the oral food challenge. Participants rated their pain on a numerical rating scale (NRS; 0 = no pain; 10 = worst pain imaginable).

The course of indigestion during the oral food challenge was measured at the same time points as pain: (1) before the oral food challenge; (2) 30 min after the oral food challenge; (3) 30–180 min after the oral food challenge; and (4) 4 h after the oral food challenge. To measure indigestion, 11 items were used based on typical symptoms described by patients with celiac disease or gluten allergy, including stomach pain, bloating, flatulence, nausea, diarrhea, headache, numbness in the legs or arms, abdominal fullness, rash, heartburn, and a sudden urge to use the toilet. Participants rated each item on a numerical ratings scale with scores ranging from 0 to 10 (0 = absence of symptom; 10 = strongest intensity; see Supplementary material for symptom list).

Secondary outcomes were expectations before and after receiving the instructions about the OLP. Expectation regarding the OLP treatment was measured with the Stanford Expectation of Treatment Scale (SETS) (Younger et al., 2012). This questionnaire consists of six items. Three items measure positive aspects of the treatment expectation: (1) This treatment will be completely effective; (2) My condition will be completely resolved after the treatment; and (3) I have complete confidence in this treatment. Three items measure negative aspects of treatment expectation: (1) I am worried about my treatment; (2) I have fears about this treatment; and (3) I am nervous about negative effects of this treatment. Participant could rate their agreement on a 7-point Likert scale (1 = do not agree at all; 7 = completely agree). Negative and positive aspects of expectation were analyzed separately. Further, expected pain relief was measured using the question, “How much pain do you expect after taking the OLP on a scale of 0–10?”

### 2.6 Statistical analysis

Analyses were conducted with IBM SPSS Statistic version 29.0 (IBM Corp., Armonk, NY, USA). A power analysis with G*Power with an expected small to medium effect size of 0.4–0.5, four measurement points and four groups, alpha = 0.05, and a power of 0.95 yielded a sample size of 64 to 96 patients. The group characteristics were compared using chi-squared tests for categorical parameters or Tukey’s test. As mentioned above, the study was originally planned exclusively with fibromyalgia patients. Therefore, sample size calculation was conducted without considering the type of disease as further group factor.

Statistical analysis was made with a stepwise approach to deal with the adjustment of the design during the course of the study. First step was the analysis of the subsample FMS only.

A repeated-measures analysis of variance (ANOVA) was conducted with “time course” as the repeated factor to determine differences in expectation, pain, and indigestion before and after the oral food challenge from time point T0 to T3. If deviations from sphericity occurred, Greenhouse–Geisser or Huynh–Feldt correction was used. If main or interaction results were significant, *post-hoc* analyses were derived using paired and independent *t*-tests, to control for multiple comparisons, a Bonferroni-correction was applied. For all analyses, two-sided *p* values of *p* < 0.05 were considered statistically significant.

To explore the relation between expectation and pain reduction, correlations were computed as Pearson’s *r*.

*Post-hoc* explorative analyses was conducted as second step to investigate the additional subgroups. As the power analysis was conducted without consideration of different pain diseases, the results of these analyses were treated as explorative approach with hypotheses-generating purpose rather than hypotheses- confirming. For patients with IBS, a repeated-measures analysis of variance (ANOVA) was conducted with “time course” as the repeated factor to determine differences in expectation, pain, and indigestion before and after the oral food challenge. If deviations from sphericity occurred, Greenhouse–Geisser or Huynh–Feldt correction was used. If main or interaction results were significant, *post-hoc* analyses were derived using a *t*-test. For all analyses, two-sided *p* values of *p* < 0.05 were considered statistically significant.

Due to the small sample size of headache patients (*n* = 6), no statistical tests were conducted. Instead, the data are presented descriptively to provide an overview of potential trends, that is generated from participants with headache.

## 3 Results

### 3.1 Participants (all pain diseases)

A total of *N* = 73 patients were included in the study and randomly divided into four groups (*n* = 18–19). Of the 73 patients, 41 (56%) were diagnosed with abdominal complaints like irritable bowel syndrome, 26 patients (36%) had FMS, and 6 patients (8%) had headache in terms of migraine or tension headache. Further, 89% were female, and the mean age of the sample was 41 (SD 14.8). The groups did not differ regarding sociodemographic variables (Table 1).

**Table 1:** Demographic baseline characteristics.

Group variable	Sham gluten, neutral instruction (*n* = 18)	Sham gluten, positive instruction (*n* = 18)	Gluten, neutral instruction (*n* = 18)	Gluten, positive instruction (*n* = 19)	*p*
Age	35.78 ± 12.1	40.33 ± 13.1	45.22 ± 15.4	43.0 ± 17.6	0.26
Sex female no. (%)	15 (83)	16 (89)	16 (89)	18 (95)	0.75
Disease per group (*N*)					
Fibromyalgia	6	6	8	6	0.95
Headache	1	2	2	1	
Irritable bowel syndrome	11	10	8	12	
Opinion gluten	3.2 ± 0.8	3.2 ± 0.5	3.1 ± 0.4	3.3 ± 0.5	0.71
Average pain before	4.4 ± 1.6	7.5 ± 12.7	4.97 ± 2.3	4.5 ± 2.2	0.50
GAMQ^1)^	3.1 ± 0.3	3.2 ± 0.3	3.2 ± 0.3	3.2 ± 0.3	0.81
SETS^2)^					
Positive expectation (before instructions)^3)^	3.44 ± 1.2	4.04 ± 0.9	4.02 ± 1.2	3.44 ± 1.1	0.16
Negative expectation (before instructions)^3)^	1.9 ± 1.3	1.5 ± 0.9	1.8 ± 1.0	1.8 ± 0.98	0.99

^1)^ GAMQ, General Attitude toward Medication; ^2)^ SETS, Stanford Expectations of Treatment Scale; ^3)^ 1 = strongly disagree, 7 = strongly agree.

### 3.2 Main analysis: fibromyalgia (*N* = 26)

#### 3.2.1 Primary outcome: pain

**Figure 3:**
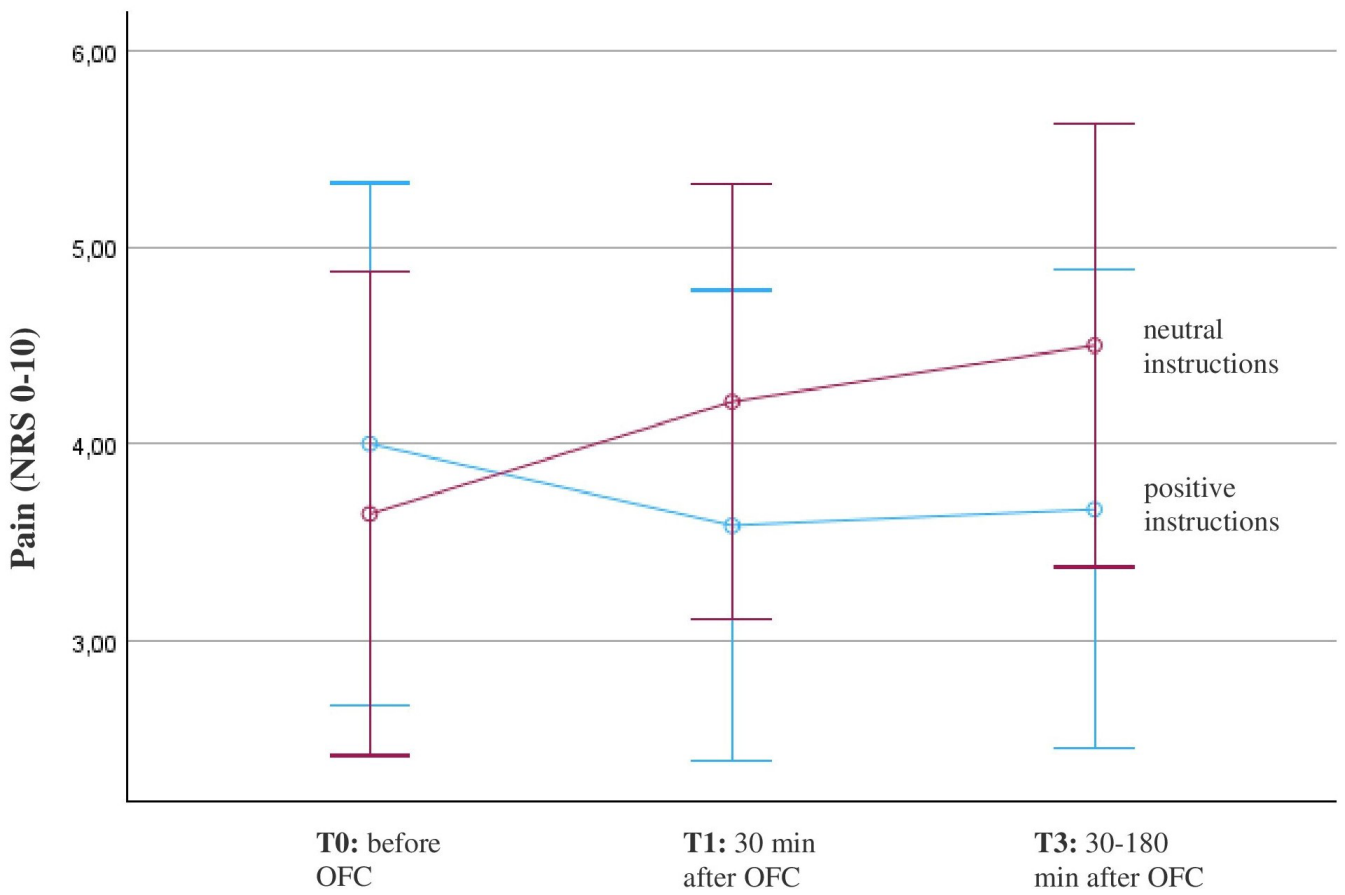
Course of pain in patients with fibromyalgia (*n* = 26) before and after the oral food challenge (OFC) and intake of Open-Label-Placebos (OLP) dependent on positive or neutral instructions about the OLP mechanism of action. Pain was measured on a numerical rating scale (NRS; 0 = no pain, 10 = worst pain). The diagram is zoomed in on the range of the y-axis, as the effects would not be visible if the full scale were used.

There were no outliers in the data of FMS patients. All groups were normally distributed, as assessed by the Shapiro–Wilk test. The course of pain during the oral food challenge depicts the primary outcome. Across all participants with FMS, there was no significant main effect *F*(1.31, 31.41) = 0.67, *p* = 0.457, *η*^2^ = 0.027. However, the repeated-measures ANOVA revealed a significant interaction effect with instruction from T0 to T2, *F*(1.41, 33.79) = 3.72, *p* = 0.048, *η*^2^ = 0.135. Participants who received positive instruction experienced a decrease in pain, while participants who received neutral instruction reported an increase in pain within the first three measurement time points (Figure 3). Despite the significant interaction effect in the ANOVA, the pairwise group comparisons showed no significant differences. Further, there was no significant interaction with gluten, *F*(1.2, 29.8) = 0.28, *p* = 0.65, *η*^2^ = 0.01. Both conditions, porridge with gluten and porridge with sham gluten, experienced a slight increase in pain after eating the porridge (Table 2).

**Table 2:** Patients with Fibromyalgia (FMS): mean values and standard deviation for pain, indigestion and expectation (*N* = 26).

Time point		T0 Baseline pre OFC before instructions^4)^		T0.1 pre OFC ^5)^ after instructions		T1 post OFC (30 min)		T2 post OFC (60–180 min)		T3 post OFC (240 min)	
Group	Outcome variable	Mean	SD	Mean	SD	Mean	SD	Mean	SD	Mean	SD
Gluten + positive instructions (*N* = 6)	Pain^1)^	4.5	1.9			3.9	1.7	4.0	1.5	3.5	1.5
Sham Gluten + positive instructions (*N* = 6)		3.5	2.3			3.3	1.2	3.3	1.2	3.2	1.5
Gluten + neutral instructions (*N* = 8)		3.6	2.7			4.3	2.5	4.8	2.6	3.6	2.8
Sham gluten + neutral instructions (*N* = 6)		3.7	2.1			4.2	2.5	4.2	2.5	4.7	3.2
Gluten + positive instructions	Indigestion^2)^	1.0	0.9			2.0	1.1	2.1	1.4	0.2	0.2
Sham Gluten + positive instructions		1.5	1.5			1.6	1.0	1.8	1.3	1.6	0.7
Gluten + neutral instructions		1.6	1.4			2.0	2.2	2.1	1.5	1.6	1.9
Sham gluten + neutral instructions		2.3	1.8			2.4	1.2	2.4	1.2	1.7	1.4
Gluten + positive instructions	Expectation – positive subscale^3) 6)^	3.5	1.3	4.2	0.7						
Sham Gluten + positive instructions		3.5	1.3	4.2	0.7						
Gluten + neutral instructions		4.1	1.2	3.6	1.6						
Sham gluten + neutral instructions		4.1	1.2	3.6	1.6						
Gluten + positive instructions	Expectation – negative subscale^3) 6)^	1.9	1.0	1.6	0.8						
Sham Gluten + positive instructions		1.9	1.0	1.6	0.8						
Gluten + neutral instructions		1.6	0.8	1.1	0.2						
Sham gluten + neutral instructions		1.6	0.8	1.1	0.2						

^1)^ 0 = no pain; 10 = worst imaginable pain. ^2)^ 0 = symptom did not occur; 10 = worst imaginable symptom. ^3)^ Items of the Stanford Expectations of Treatment Scale; 1 = do not agree at all, 7 = fully agree ^4)^ Instructions about the mechanism of action. ^5)^ OFC = oral food challenge ^6)^ as gluten was not relevant for the expectation, differences just occur between the positive and neutral instructions groups.

No significant effects *F*(2, 47) = 1.5, *p* = 0.2, *η*^2^ = 0.06 were found when all four time points were included, as pain reached the starting level after 4 h.

#### 3.2.2 Secondary outcome: expectation

To analyze the course of expectation, negative and positive aspects of treatment expectation were investigated.

##### 3.2.2.1 Positive treatment expectations

**Figure 4:**
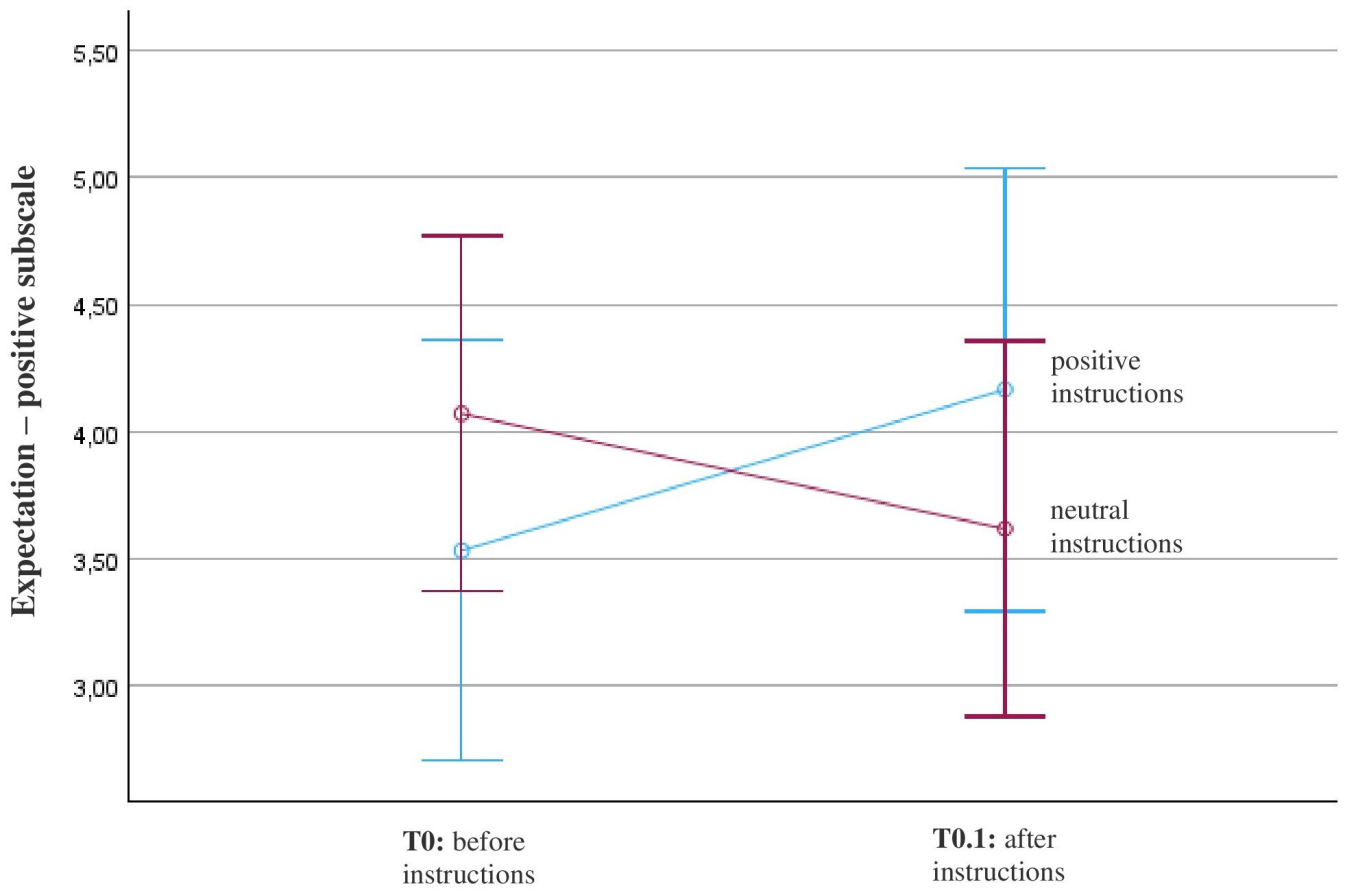
Course of positive expectation (SETS – positive subscale) in patients with fibromyalgia (*N* = 26). Patients’ expectations before and after positive or neutral instructions about the mechanism of action of OLP were measured with the positive scale of the Stanford Expectation Scale (SETS) with these items: (1) This treatment will be completely effective; (2) My condition will be completely resolved after the treatment; and (3) I have complete confidence in this treatment. The diagram is zoomed in on the range of the y-axis, as the effects would not be visible if the full scale were used (full scale = 1–7; 1 = strongly disagree, 4 = neither agree nor disagree, 7 = strongly agree).

The repeated-measures analysis of variance (ANOVA) with a Greenhouse–Geisser correction revealed that there was no statistically significant main effect of “positive expectations” before and after receiving the instructions, *F*(1, 22) = 0.1, *p* = 0.7, *η*^2^ = 0.005. However, participants who received the positive instructions indicated an increase in positive expectations, while participants who received neutral instructions indicated a decrease in positive expectations (Figure 4). This interaction effect with instructions was marginally not significant, *F*(1, 22) = 3.9, *p* = 0.06, *η*^2^ = 0.15 (Table 2).

##### 3.2.2.2 Negative treatment expectations

The repeated-measures analysis of variance (ANOVA) with a Greenhouse–Geisser correction showed that there was a statistically significant main effect of “negative expectations.” All participants with fibromyalgia experienced a decrease in negative expectations at the second measure time point in comparison to the baseline, *F*(1, 22) = 6.97, *p* = 0.01, *η*^2^ = 0.24). However, there was no significant interaction effect with “instruction,” *F*(1, 11) = 0.68, *p* = 0.4, *η*^2^ = 0.03 (Table 2).

##### 3.2.2.3 Expected pain relief

**Figure 5:**
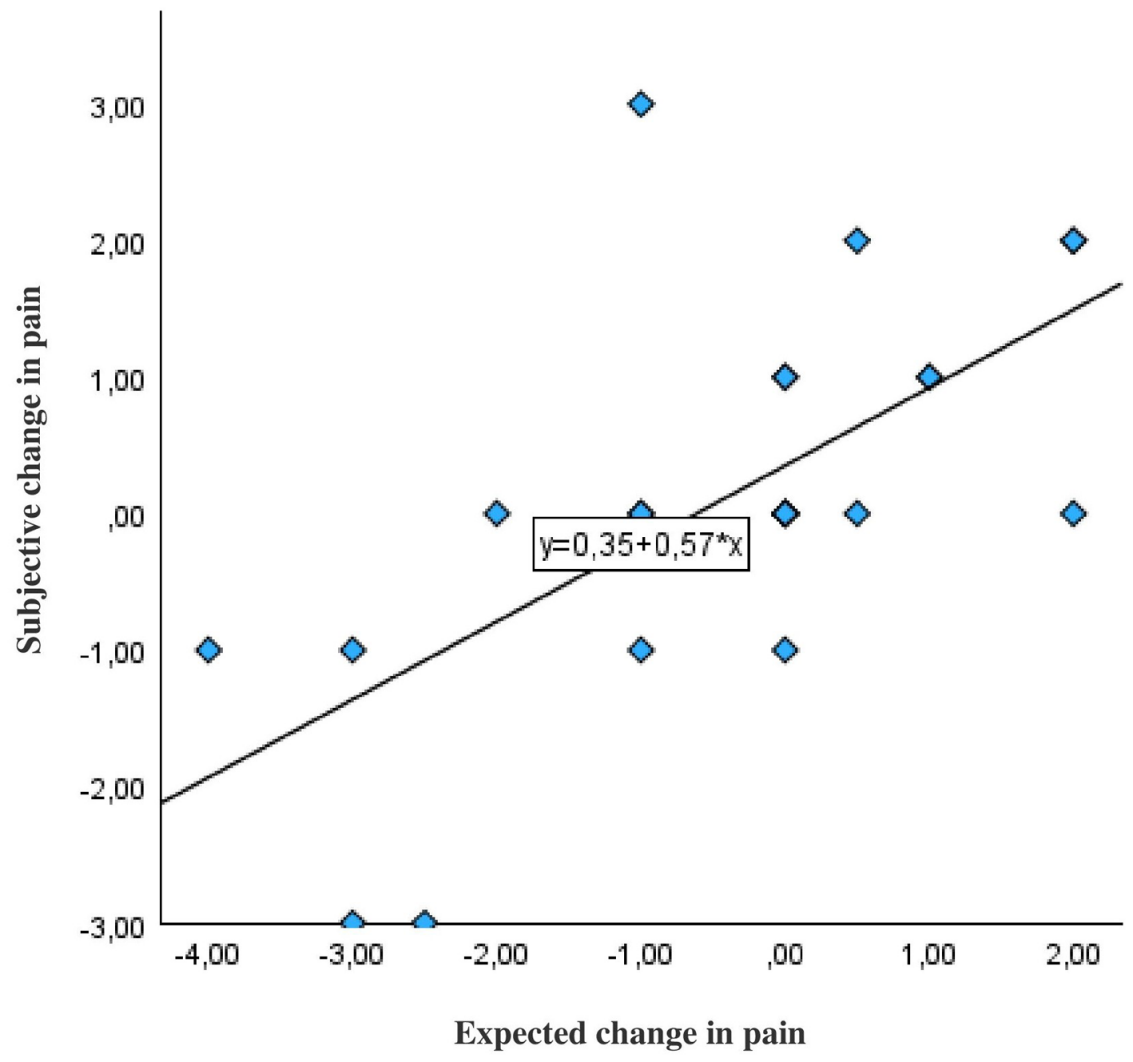
Correlation between expected and real change in pain increase or decrease before and after the instruction about the mechanism of action of OLP in patients with fibromyalgia (N = 26). The correlation is based on the real and expected difference values. Expected change in pain: Patients were asked what change in pain they believed to experience after OLP intake. Subjective change in pain: Differences in the scores of patients’ pain ratings on the numerical rating scale ranging 0–10 (10 = worst pain) were measured before and after the instructions.

Across the FMS population, there was a strong significant correlation between the expected and actual change in pain before and after the oral food challenge (Pearson’s *r* = 0.81; *p* < 0.001; Figure 5).

#### 3.2.3 Secondary outcome: indigestion

**Figure 6:**
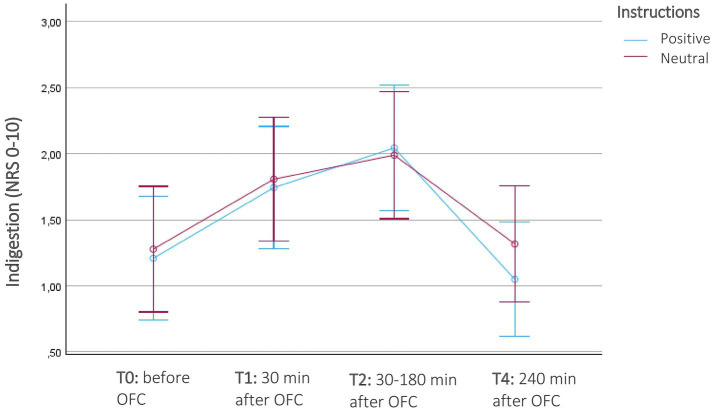
Course of indigestion in patients with fibromyalgia (*N* = 26) before and after the oral food challenge (OFC) and intake of Open-Label-Placebos (OLP) dependent on positive or neutral instructions about the OLP mechanism of action. Indigestion were measured on a numerical rating scale of 11 digestion-related symptoms (NRS; 0 = no symptom, 10 = severe symptom). The diagram is zoomed in on the range of the y-axis, as the effects would not be visible if the full scale were used.

There was a significant increase in indigestion in FMS participants before (T0) and after the OFC (T1-T3) and OLP intervention, *F*(3, 66) = 4.8, *p* = 0.004, *η*^2^ = 0.18). However, no significant interaction effect with instruction was found, *F*(3, 66) = 0.38, *p* = 0.77, *η*^2^ = 0.017. Both the positive-instruction and neutral-instruction groups experienced an increase in indigestion after eating the porridge and taking the OLP, which decreased again after 30–120 min and reached the beginning stage after 4 h (Table 2; Figure 6). Again, there was no significant interaction with the composition of the porridge, *F*(3, 66) = 1.8, *p* = 0.15, *η*^2^ = 0.08 or higher order interaction.

### 3.3 Explorative additional analyses

- Participants with IBS (*N* = 41).

#### 3.3.1 Primary outcome: pain

The repeated-measures analysis of variance (ANOVA) revealed no significant main effect of pain across time, *F*(1, 42) = 2.4, *p* = 0.1, *η*^2^ = 0.06. The neutral-instruction group experienced a pain increase, while the positive- instruction group reported a consistent pain level during the OFC. However, this interaction with instructions was not significant, *F*(1, 42) = 1.04, *p* = 0.3, *η*^2^ = 0.03. There was also no significant interaction with gluten or gluten*instructions. Both, gluten and sham gluten led to a slight increase in pain, *F*(1, 42) = 0.02, *p* = 0.7, *η*^2^ = 0.003 (Table 3).

**Table 3:** Patients with Irritable Bowel Syndrome (IBS): mean values and standard deviation for pain, indigestion and expectation (*N* = 41).

Time point		T0 Baseline pre OFC before instructions^4)^		T0.1 pre OFC ^5)^ after instructions		T1 post OFC (30 min)		T2 post OFC (60–180 min)		T3 post OFC (240 min)	
Group	Outcome variable	Mean	SD	Mean	SD	Mean	SD	Mean	SD	Mean	SD
Gluten + positive instructions (*N* = 12)	Pain^1)^	0.8	1.9			0.9	1.5	0.9	1.5	0.8	1.8
Sham Gluten + positive instructions (*N* = 10)		0.9	1.3			1.1	1.3	1.1	1.3	1.3	2.3
Gluten + neutral instructions (*N* = 8)		0.6	1.4			1.0	1.6	1.3	2.2	0.1	0.4
Sham gluten + neutral instructions (*N* = 11)		0.7	1.4			1.4	1.8	1.5	2.1	1.2	1.7
Gluten + positive instructions	Indigestion^2)^	1.5	1.6			1.8	1.6	2.5	1.9	1.5	1.5
Sham Gluten + positive instructions		1.1	1.6			1.7	1.5	1.9	1.5	0.7	1.2
Gluten + neutral instructions		0.3	0.5			0.9	0.8	1.5	1.4	0.6	0.9
Sham gluten + neutral instructions		1.5	1.4			1.7	0.8	2.0	0.7	1.5	1.5
Gluten + positive instructions (*N* = 22)	Expectation – positive subscale^3) 6)^	3.7	1.0	4.2	1.1						
Sham Gluten + positive instructions		3.7	1.0	4.2	1.1						
Gluten + neutral instructions (*N* = 19)		3.5	1.0	3.1	1.5						
Sham gluten + neutral instructions		3.5	1.0	3.1	1.5						
Gluten + positive instructions	Expectation – negative subscale^3) 6)^	1.8	1.0	1.2	0.3						
Sham Gluten + positive instructions		1.8	1.0	1.2	0.3						
Gluten + neutral instructions		1.8	1.2	1.4	0.7						
Sham gluten + neutral instructions		1.8	1.2	1.4	0.7						

^1)^ 0 = no pain; 10 = worst imaginable pain. ^2)^ 0 = symptom did not occur; 10 = worst imaginable symptom. ^3)^ Items of the Stanford Expectations of Treatment Scale; 1 = do not agree at all, 7 = fully agree ^4)^ Instructions about the mechanism of action. ^5)^ OFC = oral food challenge ^6)^ as gluten was not relevant for the expectation, differences just occur between the positive and neutral instructions groups.

#### 3.3.2 Secondary outcome: expectation

To analyze the course of expectation exploratively, negative and positive aspects of treatment expectation were investigated.

##### 3.3.2.1 Positive treatment expectations

The explorative repeated-measures analysis of variance (ANOVA) revealed that there was no statistically significant main effect of “positive expectations” before and after receiving the instructions, *F*(1, 39) = 0.003, *p* = 0.9, *η*^2^ = 0.0. However, participants who received the positive instructions indicated an increase in positive expectations, while participants who received neutral instructions indicated a decrease in positive expectations (Table 3). This interaction effect with instructions was significant, *F*(1, 39) = 4.2, *p* = 0.046, *η*^2^ = 0.98). *Post-hoc t*-tests revealed, that the post-values differed significantly between the positive and the neutral group (*p* = 0.014; 95% CI: 0.16–1.4), but not between pre and post values within the positive group (*p* = 0.1) and the neutral group (*p* = 0.2).

##### 3.3.2.2 Negative treatment expectations

There was a statistically significant change of “negative expectations.” All participants with IBS experienced a decrease in negative expectations at the second measure time point in comparison to the baseline, *F*(1, 39) = 18.2, *p* < 0.001, *η*^2^ = 0.3). *Post-hoc t*-test revealed that the difference between pre and post values were significant (*p* < 0.001; 95% CI: 0.3–1.02). However, there was no significant interaction effect with “instruction,” *F*(1, 39) = 0.7, *p* = 0.4, *η*^2^ = 0.02.

##### 3.3.2.3 Expected pain relief

Across patients with IBS, there was a moderate correlation between expected pain and actual change in pain before and after the intervention (Pearson’s *r* = 0.44; *p* = 0.004).

#### 3.3.3 Secondary outcome: indigestion

The explorative repeated- measures analysis showed that there was a significant main effect of indigestion in IBS patients. Symptoms of indigestion significantly increased at time point T1 and T2 in comparison to time point T0, *F*(3, 90) = 8.2, *p* < 0.001, *η*^2^ = 0.18. However, no significant interaction effect of instructions was observed, both groups experienced the same increase of indigestion, regardless of whether they received positive or neutral instructions, *F*(3, 90) = 0.47, *p* = 0.7, *η*^2^ = 0.012 (Table 3). There was no significant interaction effect with gluten or gluten*instructions, *F*(3, 90) = 0.6, *p* = 0.6, *η*^2^ = 0.012.

- participants with headache (*n* = 6).

Due to the small sample size, the results are presented descriptively without statistical analyses.

##### 3.3.3.1 Pain

Across all 6 patients, there was a decrease in pain between baseline (T0) M = 3 (SD = 3.5) and the last measurement point (T3) M = 0.8 (SD = 1.3). Both groups, neutral-instruction and positive-instruction reported a similar reduction in pain levels. In the positive group, the mean decrease was ∆ = −2.0, while in the neutral group, the decrease was ∆ = −2.3 (Table 4).

**Table 4:** Patients with headache: mean values and standard deviation for pain, indigestion and expectation (*N* = 6).

Time point		T0 Baseline pre OFC^5)^ before instructions^4)^		T0.1 pre OFC after instructions		T1 post OFC (30 min)		T2 post OFC (60–180 min)		T3 post OFC (240 min)	
Group	Outcome variable	Mean	SD	Mean	SD	Mean	SD	Mean	SD	Mean	SD
Gluten + positive instructions (*N* = 1)	Pain^1)^	0.0	–			0.0	–	0.0	–	0.0	–
Sham Gluten + positive instructions (*N* = 2)		3.0	4.2			2.5	3.5	0.0	0.0	0.0	0.0
Gluten + neutral instructions (*N* = 2)		2.0	2.8			1.5	2.1	1.5	2.1	1.0	1.4
Sham gluten + neutral instructions (*N* = 1)		8.0	–			7.0	–	2.0	–	3.0	–
Gluten + positive instructions	Indigestion^2)^	0.0	–			1.3	–	1.3	–	1.5	–
Sham Gluten + positive instructions		0.3	0.5			1.5	2.1	0.8	1.2	0.7	0.9
Gluten + neutral instructions		0.3	0.5			2.7	3.8	2.7	3.8	0.8	1.2
Sham gluten + neutral instructions		0.5	–			0.8	–	1.7	–	1.2	–
Gluten + positive instructions (*N* = 3)	Expectation – positive subscale^3) 6)^	4.2	0.5	4.7	0.6						
Sham Gluten + positive instructions		4.2	0.5	4.7	0.6						
Gluten + neutral instructions (*N* = 3)		3.4	0.2	4.3	0.9						
Sham gluten + neutral instructions		3.4	0.2	4.3	0.9						
Gluten + positive instructions	Expectation – negative subscale^3) 6)^	1.6	1.0	1.1	0.2						
Sham Gluten + positive instructions		1.6	1.0	1.1	0.2						
Gluten + neutral instructions		2.7	0.8	1.2	0.4						
Sham gluten + neutral instructions		2.7	0.8	1.2	0.4						

^1)^ 0 = no pain; 10 = worst imaginable pain. ^2)^ 0 = symptom did not occur; 10 = worst imaginable symptom. ^3)^ Items of the Stanford Expectations of Treatment Scale; 1 = do not agree at all, 7 = fully agree ^4)^ Instructions about the mechanism of action. ^5)^ OFC = oral food challenge ^6)^ as gluten was not relevant for the expectation, differences just occur between the positive and neutral instructions groups.

The consumption of the porridge again led to a decrease of pain in both groups, gluten and sham gluten. However, the reduction in the sham gluten group (∆ = −3.6) was larger than in the gluten group (∆ = −0.6).

##### 3.3.3.2 Indigestion

The type of instructions did not lead to different courses of indigestion. Both groups indicated an increase of indigestion during the oral food challenge with a mean change of ∆ = 1.3 in the positive group and ∆ = 1.7 in the neutral group.

Both group, gluten and sham gluten, showed an increase in digestive discomfort. However, headache patients who received real gluten indicated a higher increase (∆ = 2) than patients who received sham gluten (∆ = 0.9).

##### 3.3.3.3 Expectation

Positive treatment expectation: both groups, positive instructions and neutral instructions, reported an increase at the subscale for positive treatment expectation (Table 4). The positive-instruction group indicated a mean change of ∆ = 0.5, while the neutral-instruction group indicated a mean change of ∆ = 0.9.

Negative treatment expectation: a decrease of negative treatment expectation was observed in in both groups, independently of the type of instruction. There was a mean change of ∆ = −0.6 in the positive-instruction group and ∆ = −1.6 in the neutral-instruction group.

## 4 Discussion

This study investigated the effects of different instructions (positive vs. neutral) for an open-label treatment and those of an active substance (gluten) or sham gluten on chronic pain patients’ pain experience (primary outcome) and on treatment expectations and subjective symptoms of wheat intolerance (secondary outcomes). Different groups of chronic pain patients were involved: (1) patients with FMS; (2) those with chronic headache; and (3) patients with irritable bowel syndrome.

Patients participating in our study underwent a double-blinded gluten provocation. Open-Label-Placebos (OLP) with positive or neutral instructions were used to influence pain and indigestion.

The major findings of the initial sample design (FMS patients) of this study are as follows: (1) There is a trend, that positive instructions lead to more positive treatment expectations regarding the Open-Label-Placebos in FMS patients; (2) A positive treatment expectation regarding the Open-Label-Placebos led to pain decrease in patients with FMS; (3) Treatment expectation served as a predictor of pain increase or decrease; (4) Open-Label-Placebos, regardless of whether patients were positively or neutrally instructed, did not influence indigestion; (5) However, also the gluten in the porridge did not affect pain nor indigestion.

(1) It is known from placebo research with concealed placebos that positive instructions lead to positive expectations regarding the subsequent treatment (Schmitz et al., 2019; Klinger et al., 2017; Solle et al., 2021; Smits et al., 2021). Research on positive expectations of OLPs is still scarce. In our study, significance of the interaction with instruction was marginally missed in participants with FMS. However, in an exploratory analyses of the IBS subgroup, we found a significant interaction with positive and neutral instructions on patients’ treatment expectations of the OLP. This result is in line with Locher et al. (2017), who found OLP administered with a rationale for effectiveness to be superior to OLP administered with a neutral rationale (Locher et al., 2017). In contrast, Schaefer et al. (2018) did not find a difference between providing extended information and offering no information (Schaefer et al., 2018). However, in their study, no manipulation check was made; hence, it is not clear whether the extended information actually led to a change in expectation, as the researchers did not measure expectation. In our study, however, the observed effects of treatment expectation served as a manipulation check and showed that changes in pain are indeed due to changes in expectation.

Previous placebo studies have used a wide range of different types of explanations, underlining different aspects of placebo mechanisms (Schmitz et al., 2019; Klinger et al., 2017; Carvalho et al., 2016; Hoenemeyer et al., 2018; Kaptchuk et al., 2010). Despite some evidence that participants prefer to hear explanations regarding brain mechanism regarding placebo effects (Smits et al., 2021), there is no clear suggestion of a certain explanation that leads to higher placebo effects (Smits et al., 2021). In general, instructions seem to explain just a small variance of placebo effects (Smits et al., 2021). Instead, observed placebo effects could also arise from a combination of instructions and the experimenter’s expectation through subtle non-verbal behavior. Daniali et al. (2024) found that positive information about a dental procedure did not lead to less pain when its administration was unknown to the treatment team. They concluded that the provision of positive information by the treatment team itself may lead to subtle non-verbal behaviors that contribute to the placebo effect. The role of these non-specific factors (patient-practitioner relationship, warmth, facial expressions, etc.) in treatment outcome has also been investigated by several other studies (Daniali and Flaten, 2019; Chen et al., 2019; Kaptchuk et al., 2008).

The present study did not take these factors into account, and it cannot be ruled out, that the behavior and characteristics of the experimenter explain some of the change in treatment expectation. For example, it is also not unlikely that the positive instructions were delivered with more enthusiasm because of the experimenter’s expectation.

However, the use of OLP may represent a special case regarding the information provided. The aforementioned studies used treatments that are regularly used in clinical settings (dental procedures, creams, acupuncture). OLP, on the other hand, is a new treatment approach where participants cannot refer to previous experience or common sense. Therefore, information about the treatment may be more important for innovative treatments, especially when openly administered placebos are used.

How expectations are measured is an important aspect for assessing the influence of instructions on treatment expectations of OLP interventions. In our study, we found that positive and negative treatment expectations differed and were not manifestations of a single dimension. A strength of the SETS questionnaire is that it includes both positive and negative expectations regarding the treatment. Studies show that positive and negative aspects of expectation are examples of two distinct dimensions (Laferton et al., 2017; Stuhlreyer and Klinger, 2022). In our study, positive instructions only changed positive expectations, but had no influence on potential negative expectations.

Instead, our results show that participants of all subgroups do not worry a lot about using OLP or about potential side effects, regardless of the instructions they are given (Tables 2–4). They indicate strong disagreement regarding negative aspects of expectation, such as side effects or concerns about OLP. However, this was even more pronounced at the second measurement point, which might indicate that even small doubts could be reduced over time. One possible explanation could be that the participants’ contact with the researcher provided additional security and confidence even without them being provided with extended information on the OLP.

(2) Our second finding was that a positive treatment expectation regarding Open-Label-Placebos lead to pain decrease in patients with FMS but not in patients with IBS or headache.

Originally the study was planned only with FMS patients. In this subgroup a significant interaction effect with the type of instructions could be found. In *post-hoc* tests differences did not reach significance probably due to the small sample size and effect size. The main purpose of this study was to better understand expectation related psychological processes of pain development in connection with gluten consumption. Due to recruiting difficulties, further pain conditions were included in the assumption that the subjective pain-gluten connection would serve as main criteria to detect significant effects. However, according to the analyses, this may have been a misjudgment, as differences between the pain diseases were found, although this was not the purpose of the study.

Significant interaction with instructions was only found in patients with FMS. Although, a similar trend could be observed in patients with IBS, this did not reach significance. Fibromyalgia and irritable bowel syndrome have some characteristics in common, such as a heightened pain sensitivity, sleep disturbance, and fatigue, and both are treated with similar medical approaches, such as the use of antidepressants (Chalaye et al., 2012). Chalaye et al. (2012) suggest that dysfunctions in descending pain inhibition play a role in both irritable bowel syndrome and fibromyalgia. However, this phenomenon appears to be more pronounced in patients with FMS (Chalaye et al., 2012), especially after long-term exposure to FMS pain (Kosek et al., 2017). One factor that could have influenced the results is the possibility of a floor effect in patients with IBS. IBS patients did not necessarily suffer from pain at the start of the experiment. Therefore, the placebo intervention could at most limit the increase in pain but could not actually reduce pain—as was the case with the FMS patients who had pain before the OFC.

In patients with headache, different response patterns could be observed, as all of them experienced a pain decrease, independent of gluten or the instructions. However, these observations cannot be generalized due to the small sample size. In our study, participants with headache included both those with migraine and those with tension headache. It is well known that despite some similarities, migraine and tension headache are distinct pain disorders with different underlying pathophysiological mechanisms (Onan et al., 2023). Especially the role of food seems to play a different role between migraine and tension headache (Haque et al., 2012). Furthermore, compared to cases of fibromyalgia and irritable bowel syndrome, gluten is rarely mentioned as a possible trigger for increased pain in patients with tension headaches.

Our results further show that (3) expectation served as a predictor of pain increase or decrease in patients with FMS and IBS. A recent meta-analysis (Buergler et al., 2023) has confirmed that at least some level of expectation enhances the effect of OLP. However, not all studies found expectation to be correlated with treatment results, and it is reasonable to believe that expectation and conditioning might at least not be the only mechanism important in OLP interventions (Schaefer et al., 2018; Kaptchuk, 2018).

Further, in our study, (4) OLP could influence indigestion neither in patients with irritable bowel syndrome nor in patients with fibromyalgia. In another study, placebos were indeed able to improve the symptoms of IBS patients, including indigestion (Kaptchuk et al., 2010). Our whole study took place in a pain center of a clinic, with the focus being on pain medicine and pain psychology. It is possible that the IBS patients were not able to identify 100% with the study and the context, which weakened the placebo effects in indigestion. The main focus of the study and the rationale behind the placebos was pain management. Although the rationale behind the OLP referred to both placebo analgesia and reduction of indigestion, it may be that the focus majorly lay on pain. An OLP intervention with more than one objective could lead to expectation effects not being sufficiently focused on one of the processes. As there are different physiological processes involved in pain and indigestion, it may be better to focus on one treatment outcome. Visceral pain, for instance, has been shown to have a higher emotional component than cutaneous pain and activates different brain areas (Strigo et al., 2002). In general, studies investigating placebo effects in gastrointestinal diseases other than IBS are rare. There are some studies that point to possible placebo effects in lactose intolerance (Briet et al., 1997; Vernia et al., 2010); however, a detailed investigation of placebo effects in this area is lacking. One narrative review points out that placebo effects in functional gastrointestinal disorders are not significantly higher than in other functional and organic diseases (Enck and Klosterhalfen, 2020).

Our last finding was that (5) the presence of gluten in the porridge did not have a significant role in the development of pain or indigestion. This result supports the current belief that there might be a high proportion of people undergoing nocebo effects when reporting gluten intolerance (de Graaf et al., 2024), even if one cannot completely rule out that gluten may have an effect on some patients. For clinical application, this means that patients must be informed about the possible nocebo effect. This could be the basis for providing special psychological interventions for this group of patients.

### 4.1 Limitations

Our study is the first to investigate expectation effects in pain patients undergoing a gluten challenge through using openly administered placebos.

However, some limitations of the study should be pointed out.

First, although all participants suffered chronic pain, the sample was still composed of a quite heterogeneous group with four different pain diseases. As mentioned above, other pain conditions were added during the course of the study to ensure that the study could be conducted. Therefore, this factor was not included in the sample size calculation, which explains the power issues and is a major limitation of the study. Although the pain conditions differ in their pathophysiology, it was not hypothesized that they would differ in their expression of response, which seems to be a misjudgment. Additionally, the majority of participants believed to be sensitive to gluten, but not all, which could have influenced the results.

Further, although we found a significant difference in expectations, the use of the SETS questionnaire in the study of OLP does not seem to be the perfect choice, especially in chronic pain patients. For instance, item 2— “My condition will be completely resolved after the treatment”—seems to be unrealistic in a patient with a chronic condition. Further, item 3— “I have complete confidence in this treatment”—probably did not measure the positive aspect of expectation in our sample, because most participants interpreted this item in terms of “not worrying that something bad will happen.” Having a clear definition of expectation and choosing the right instrument remains a challenge (Laferton et al., 2017; Bialosky et al., 2010). Especially in placebo research, this topic has to be addressed carefully.

Another limitation refers to the description of the instructions as ‘positive’ and ‘neutral’. Participants in the neutral group received the information that “these are placebo tablets without pharmacologically active ingredients,” which is a simple definition of the term “placebo tablet.” However, it could be argued that this type of instruction is not completely neutral, as participants might be disappointed about not receiving a “real treatment.” One indication of this could be the fact that the neutral group in our study experienced a reduction in positive treatment expectations.

The final limitation refers to the lack of a control group that could have consisted of patients undergoing the food challenge without taking placebos to compare symptoms with participants who received their OLP with neutral instructions.

## 5 Conclusion

The outcomes of this study reveal that expectation effects play a pivotal role in gluten intolerance in chronic pain patients. The use of OLPs might be a promising approach to decrease pain or prevent an increase in pain after the consumption of food that is associated with pain. It could ease exposure to and the desensitization of feared food components in chronic pain patients. However, the extent to which OLP can help with indigestion regarding food intolerance is yet unclear.

## Data Availability

The raw data supporting the conclusions of this article will be made available by the authors, without undue reservation.
