# Supplementary material for: Open-Label-Placebos can reduce pain, but not indigestion during gluten challenge in chronic pain patients
Source: Front Psychol. 2025 Jun 4;16:1572761. doi: 10.3389/fpsyg.2025.1572761 (PMC12174411; doi:10.3389/fpsyg.2025.1572761)
Supplement: Supplementary file 1 [file Presentation_1.pdf]

Supplement material

Assessment of indigestion (secondary outcome) at time points T0, T1, T2, T3:

I suffer from **abdominal pain**:

(0)	(1)	(2)	(3)	(4)	(5)	(6)	(7)	(8)	(9)	(10)
no abdominal pain										severe abdominal pain

I feel **bloated:**

(0)	(1)	(2)	(3)	(4)	(5)	(6)	(7)	(8)	(9)	(10)
Not at all										very strong

I suffer from **outgoing flatulences**:

(0)	(1)	(2)	(3)	(4)	(5)	(6)	(7)	(8)	(9)	(10)
Not at all										very strong

I suffer from **nausea:**

(0)	(1)	(2)	(3)	(4)	(5)	(6)	(7)	(8)	(9)	(10)
Not at all										very strong

I suffer from **diarrhea:**

(0)	(1)	(2)	(3)	(4)	(5)	(6)	(7)	(8)	(9)	(10)
Not at all										very strong

I suffer from **headache:**

(0)	(1)	(2)	(3)	(4)	(5)	(6)	(7)	(8)	(9)	(10)
Not at all										very strong

I suffer from **joint pain:**

(0)	(1)	(2)	(3)	(4)	(5)	(6)	(7)	(8)	(9)	(10)
Not at all										very strong

I suffer from **muscle pain:**

(0)	(1)	(2)	(3)	(4)	(5)	(6)	(7)	(8)	(9)	(10)
Not at all										very strong

My **arms** and **legs** feel **numb:**

(0)	(1)	(2)	(3)	(4)	(5)	(6)	(7)	(8)	(9)	(10)
Not at all										very strong

I suffer from **abdominal fullness:**

(0)	(1)	(2)	(3)	(4)	(5)	(6)	(7)	(8)	(9)	(10)
Not at all										very strong

I suffer from **skin rashes:**

(0) (1) (2) (3) (4) (5) (6) (7) (8) (9) (10)
Not at all very strong

I suffer from **heartburn:**

(0) (1) (2) (3) (4) (5) (6) (7) (8) (9) (10)
Not at all very strong

I have the **sudden urge** to go to the toilet:

(0) (1) (2) (3) (4) (5) (6) (7) (8) (9) (10)
Not at all very strong
